# Detection of gene pathways with predictive power for breast cancer prognosis

**DOI:** 10.1186/1471-2105-11-1

**Published:** 2010-01-01

**Authors:** Shuangge Ma, Michael R Kosorok

**Affiliations:** 1 School of Public Health, Yale University, New Haven, CT 06520, USA; 2 Department of Biostatistics, University of North Carolina, Chapel Hill, NC 27599, USA

## Abstract

**Background:**

Prognosis is of critical interest in breast cancer research. Biomedical studies suggest that genomic measurements may have independent predictive power for prognosis. Gene profiling studies have been conducted to search for predictive genomic measurements. Genes have the inherent pathway structure, where pathways are composed of multiple genes with coordinated functions. The goal of this study is to identify gene pathways with predictive power for breast cancer prognosis. Since our goal is fundamentally different from that of existing studies, a new pathway analysis method is proposed.

**Results:**

The new method advances beyond existing alternatives along the following aspects. First, it can assess the predictive power of gene pathways, whereas existing methods tend to focus on model fitting accuracy only. Second, it can account for the joint effects of multiple genes in a pathway, whereas existing methods tend to focus on the marginal effects of genes. Third, it can accommodate multiple heterogeneous datasets, whereas existing methods analyze a single dataset only. We analyze four breast cancer prognosis studies and identify 97 pathways with significant predictive power for prognosis. Important pathways missed by alternative methods are identified.

**Conclusions:**

The proposed method provides a useful alternative to existing pathway analysis methods. Identified pathways can provide further insights into breast cancer prognosis.

## Background

Amongst women in the US, breast cancer is the most commonly diagnosed malignancy after skin cancer, and is the second leading cause of cancer deaths after lung cancer. According to the American Cancer Society, in 2009, an estimated 192,370 new cases of breast cancer were diagnosed, and 40,610 died from breast cancer. Women in the US have a 1 in 8 lifetime risk of developing invasive breast cancer and a 1 in 33 overall chance of dying from it. Biomedical studies suggest that genomic measurements may have independent predictive power for breast cancer prognosis [1,2].

Multiple gene profiling studies have been conducted, searching for genomic measurements with predictive power for breast cancer prognosis. "Breast cancer has probably been the carcinoma most intensively studied by gene expression profiling" [1]. In this article, when referring to "prognosis", we limit ourselves to relapse-free survival. The overall and other types of survival have different patterns and different genomic bases, and need to be investigated separately. Examples of gene expression profiling studies on breast cancer prognosis include [3], which used Affymetrix U133A microarrays and identified 97 genes including UBE2C, KPNA2, TPX2, FOXM1, STK6, CCNA2, BIRC5, and MYBL2. Ivshina et al. [4] reported similar findings from a concurrent, independent study. Researchers at the Netherlands Cancer Institute identified a 70-gene prognostic signature [5]. Many genes involving the hallmarks of cancer were included: cell cycle, metastasis, angiogenesis, and invasion. This gene signature was then validated on an independent cohort of 295 patients [6]. References to more studies can be found in [1,2].

When searching for genomic measurements with predictive power for breast cancer prognosis, it is necessary to account for the inherent coordination among genes. Such coordination can be described with the pathway structure, where pathways are composed of multiple genes with coordinated biological functions.

In cancer genomic studies, tremendous effort has been devoted to pathway based analysis. "Pathway analysis is a promising tool to identify the mechanisms that underlie diseases, adaptive physiological compensatory responses, and new avenues for investigation" [7]. Compared with individual gene based analysis, pathway based analysis may lead to results that are more reproducible and more interpretable. Examples of pathway analysis methods include the gene set enrichment analysis (GSEA) [8], the Globaltest approach [9], the Maxmean approach [10], and others. We refer to [11-13] for comprehensive reviews on the subject.

Consider a pathway composed of *m *genes. Denote *X *= (*X*^1^, ..., *X*^*m*^)' as the gene expressions. Consider breast cancer relapse-free survival. We refer to the "Methods" section for detailed descriptions of the data and model setup. Determining the predictive power amounts to determining whether there exists a length-*m *vector *β *such that *β*'*X *can be used to separate patients into groups with different survival risks. We first note that,

(a) Different pathways have different biological functions. Thus, it is reasonable to study each pathway separately. Among the many pathways, only a few have predictive power for cancer development. Among genes within predictive pathways, there are a subset having small to moderate predictive power, whereas the remainder are "noisy" genes. Within each pathway, instead of investigating each *X*^*i *^separately (i.e, the *marginal *effect of each gene), it is more sensible to study *β*'*X *(i.e, the *joint *effects of multiple genes);

(b) Cancer genomic studies often have small sample sizes, and sizes of gene pathways can be large. When investigating the joint effects of multiple genes in a pathway, if the same dataset is used for estimation of *β *as well as evaluation of predictive power, the evaluation can be seriously biased [14].

Ideally, there should be two independent datasets: a training set and a testing set. *β *should be generated using only subjects in the training set. Then predictions can be made for subjects in the testing set using the training set estimate, and the predictive power can be evaluated.

Although there are many existing pathway analysis methods, they are not suitable for detecting predictive gene pathways for one or more of the following reasons. (a) For a specific pathway, they analyze each gene separately, and then draw conclusions on the pathway by combining results on individual genes. Such methods, including the GSEA and Maxmean, are suitable for answering "which pathways are enriched with genes that are *marginally differentially expressed"*. They cannot quantify the *joint effects of genes *in a pathway; (b) They focus on the model fitting aspect of genes, as opposed to prediction. When studying one or a small number of genes, model fitting performance can be a reasonable proxy for prediction performance. However, when investigating a moderate to large number of genes, because of the possibility of overfitting, model fitting performance can be a biased proxy for prediction; and (c) They analyze only a single dataset. Cancer genomic studies have small sample sizes and a large number of gene expressions. Results obtained from analysis of a single dataset may lack reliability [15].

In this article, we propose a new method for detection of predictive gene pathways. It has the following desirable features. (a) For each pathway, it uses a single statistical model to describe the effects of all genes in the pathway. Thus, it can account for the joint effects of genes; (b) A penalized approach is used to construct *β*. The penalized approach can carry out regularized estimation and gene selection simultaneously. Adopting the penalized approach has been motivated by the following considerations. First, when the pathway sizes are larger than or comparable to the sample size, the penalized approach can effectively avoid overfitting. Second, even in a predictive pathway, there may still exist noisy genes. The penalized approach can separate predictive genes from noisy ones and use only predictive genes in the statistical models. This can lead to better performance than using all the genes; (c) A random partition is used to split data into a training set and a testing set. Ideally, the training and testing sets should come from independent studies. However, for most cancer genomic studies, it can be difficult to find studies with a comparable design. For example, different studies may use different platforms for profiling. Estimates generated from a dataset using cDNA cannot be directly used for prediction for a dataset using Affymetrix. To make the proposed method broadly applicable, we use random partitions to "generate" independent datasets. To avoid an extreme partition, we will carry out multiple partitions; (d) The proposed method can analyze multiple datasets and generate results that are more reliable than analysis of a single dataset.

## Results and Discussion

### Data collection and processing

Shen et al. [16] collected data from four breast cancer prognosis studies, evaluated their designs, and concluded that they are comparable and can be pooled for meta analysis. In this study, we analyze the same four datasets. Of note, Shen et al. [16] and the present study focus on individual genes and gene pathways respectively. Thus, results from the two studies are not directly comparable.

We provide brief descriptions of the four studies in Table 1, and refer to the original publications for more detailed information. Among the four datasets, two used cDNA, one used oligonucleotide arrays, and one used Affymetrix GeneChips for profiling. Considering the incomparability of different profiling techniques, we cannot straightforwardly combine the four datasets. Neither can we use estimates from one dataset to make predictions for subjects in another set. We refer to [15] for more discussion on this issue.

**Table 1:** Breast cancer prognosis studies.

Reference	Platform	Gene	Sample
Sorlie et al. [47]	cDNA	8102	58
van't Veer et al. [5]	Oligonucleotide	24481	78
Huang er al. [48]	Affymetrix	12625	71
Sotiriou et al. [17]	cDNA	7650	98

Platform: platforms used for profiling; Gene: number of gene expressions measured; Sample: sample size.

We process each dataset separately as follows. We conduct microarray normalization using a lowess normalization for cDNA data and a robust normalization for Affymetrix data. We impute missing measurements using the k-nearest neighbors approach. We then normalize gene expressions to have zero median and unit variance.

We match genes in the four studies using their Unigene Cluster IDs, and identify 2555 genes that are measured in all four studies.

### Construction of gene pathways

For each gene, we search KEGG http://www.genome.ad.jp/kegg/ for its pathway information. Only genes belonging to known pathways are used in downstream analysis. Since breast cancer prognosis is studied, we pay special attentions to "cancer-related" pathways http://www.sonycsl.co.jp/person/tetsuya/sub2.html. Among the 2555 genes, 711 belong to 169 KEGG pathways. The pathway sizes range from 1 to 51, with median size 7.

### Detection of predictive pathways

When implementing the proposed method, we select the tuning parameter *λ*_*n *_using 3-fold cross validation. We set the bridge penalization parameter to *γ *= 1/2. For each dataset and each pathway, *B *= 100 random partitions are employed to compute the Observed Predictive Index (OPI) and Permuted Predictive Index (PPI) which are defined in the "Methods" section. In the multiple comparison adjustment, we set the target false discovery rate to *q *= 0.2. We refer to the "Methods" section for detailed descriptions of the aforementioned parameters and measurements.

**Figure 1:**
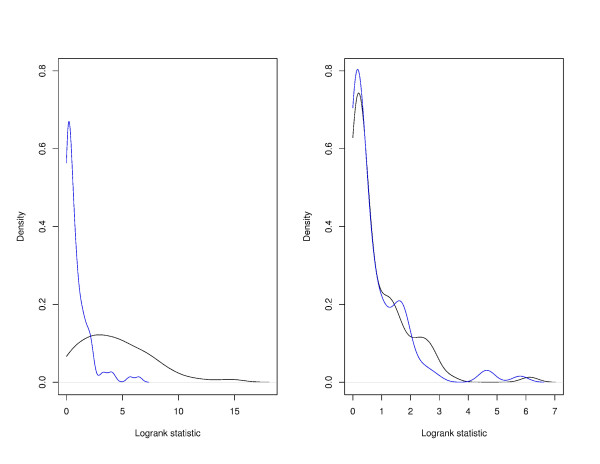
**Densities of OPI and PPI**. Left panel: the Dentatorubropallidoluysian atrophy pathway, which has predictive power; Right panel: the Thyroid cancer pathway, which does not have predictive power. Black line: density of OPI; Blue line: density of PPI. Data from [17].

With the proposed method, we use the separation of OPI and PPI to measure the predictive power. To gain more insight, we show representative plots of the OPI and PPI in Figure 1. For the dataset described in [17], we select two pathways - the Dentatorubropallidoluysian atrophy pathway which contains 5 genes and is identified as predictive, and the Thyroid cancer pathway which also contains 5 genes and is not predictive. For a better visualization, we plot the estimated densities, rather than histograms, in Figure 1. We can see that for the predictive pathway (left panel), the OPI and PPI are well separated. However, for the pathway without predictive power (right panel), the OPI and PPI are almost completely overlapped.

96 pathways are identified as having predictive power for breast cancer prognosis. Those pathways have sizes ranging from 1 to 51, with median size 7. We provide detailed information, including pathway name, size, and unadjusted p-value, on the top 20 pathways in Table 2, and on all the identified pathways in the Additional File 1.

**Table 2:** Top 20 pathways identified using the proposed approach.

Pathway	Size	P-value
Glutamate metabolism	5	2.22E-16
Amyotrophic lateral sclerosis (ALS)	6	5.55E-15
Colorectal cancer	19	2.26E-13
Small cell lung cancer	27	1.15E-12
Streptomycin biosynthesis	2	1.95E-12
Cell cycle	36	3.10E-12
Prion disease	4	3.37E-12
Renin-angiotensin system	2	8.99E-12
Nicotinate and nicotinamide metabolism	5	1.13E-11
Circadian rhythm	2	4.24E-11
Glycerophospholipid metabolism	14	4.36E-11
Prostate cancer	20	1.21E-10
Apoptosis	27	4.67E-10
Oxidative phosphorylation	15	7.32E-10
Synthesis and degradation of ketone bodies	2	8.75E-10
D-Glutamine and D-glutamate metabolism	2	1.78E-09
Focal adhesion	49	2.07E-09
Dentatorubropallidoluysian atrophy (DRPLA)	5	2.24E-09
Renal cell carcinoma	17	5.06E-09
Neurodegenerative Disorders	10	5.56E-09

Size: number of genes in the pathway; p-value: unadjusted p-value.

The glutamate metabolism pathway has the smallest unadjusted p-value. It contains five genes: GLUD1, GSS, GCLM, CAD, and glutaminase. Glutamate is a central junction for interchange of amino nitrogen. It facilitates both amino acid synthesis and degradation. The metabotropic glutamate receptors (Grm) mediate a diverse array of cellular signaling responses including hormone, neurotransmitter, chemokine, autocrine, and paracrine factors. Grm over-expression has been observed in several malignancies. Gorski et al. [18] described this over-expression of Grm in invasive breast cancer. Among the five genes in the Glutamate metabolism pathway, interrogation of the Comparative Toxigenomics Database [19] suggests that four of them (all but gene CAD) have been previously identified as breast cancer susceptibility genes.

The pathway with the second highest significance is the Amyotrophic lateral sclerosis (ALS) pathway, which contains six genes: PPP3CA, KARS, CAT, RAB5A, GPX1, and BCL2. Searching the Comparative Toxigenomics Database suggests that all six genes have been previously identified as associated with breast cancer prognosis. Of special interest are gene RAB5A, which is a member of the RAS oncogene family, and gene CAT, which has been identified to be associated with breast cancer via multiple channels.

We have also examined the biological functions of other identified pathways, and found that many of them have independent evidences of being associated with breast cancer prognosis. In particular, among the top 20, a few of them are known hallmarks of cancer, including the cell cycle pathway (36 genes; rank 6), apoptosis pathway (27 genes; rank 13), D-Glutamine and D-glutamate metabolism pathway (2 genes; rank 16), and focal adhesion pathway (49 genes; rank 17). In addition, among the pathways ranking 21-76, many have been established as having predictive power, including the VEGF signaling pathway, Ribosome, MAPK signaling pathway, Insulin signaling pathway, Wnt signaling pathway, DNA polymerase, and others. The sound biological basis of identified pathways partly validates the proposed method.

Among the 73 pathways identified as not having predictive power is the ErbB signaling pathway, which contains 16 genes. Gene ErbB2 is an oncogene and has been identified as associated with breast cancer.

There are multiple possible explanations for why the proposed method does not identify the ErbB signaling pathway, including for example limitations of the proposed method and the limited data analyzed. Of note, this pathway cannot be identified using any of the alternatives considered in the next subsection.

Interrogation of the remaining 72 pathways does not suggest any obvious false negatives.

### Analysis with alternative methods

To provide a more comprehensive understanding of the proposed method, we also analyze the same data using the following three alternatives. With each alternative method, we first analyze each dataset separately, and then conduct meta analysis following a procedure similar to the one described in the "Methods/Meta analysis" subsection.

#### Gene set enrichment analysis

With the GSEA [8], 16 pathways are identified, 7 of which are also identified with the proposed method. Detailed information is provided in the Additional File 1. Among the top 20 pathways identified using the proposed method, the GSEA does not identify any.

#### Maxmean method

3 pathways are identified, all of which are identified by the proposed method. Among the top 5 pathways identified using the proposed method, the Maxmean does not identify any. Among the top 20, the Maxmean identifies 2. Detailed information on Maxmean identified pathways is provided in the Additional File 1.

#### Globaltest method

With the Globaltest [9], 78 pathways are identified, 61 of which are also identified by the proposed method. More detailed information is provided in the Additional File 1. Among the top 5 pathways identified using the proposed method, the Globaltest misses the Streptomycin biosynthesis pathway (rank 5; size 2). Among the top 20 pathways, the Globaltest also misses the Nicotinate and nicotinamide metabolism pathway (rank 9; size 5) and the Dentatorubropallidoluysian atrophy pathway (rank 18; size 5). Although at first look, these three pathways do not seem to be directly linked with breast cancer prognosis, interrogation of NCBI and CTD [19] suggests that they in fact contain important, established breast cancer markers.

Specifically, the Streptomycin biosynthesis pathway contains two genes: PGM1 (Phosphoglucomutase 1) and IMPA2 (Inositol(myo)-1(or 4)-monophosphatase 2), which are involved in the metabolism of carbohydrate, glucose, inositol, and phosphate. Phosphoglucomutases (PGM) catalyze the transfer of phosphate between the 1 and 6 positions of glucose. In most cell types, PGM1 isozymes predominate, representing about 90% of total PGM activity. This gene has been identified as one of the ER status markers in the diagnosis and prognosis of breast cancer patients [20]. Gene IMPA2 is also associated with ER status in breast cancer patients [21] and with breast cancer metastasis to bone [22]. It is one of the breast cancer markers in the Genes-to-Systems Breast Cancer Database [23].

The Nicotinate and nicotinamide metabolism pathway contains five genes: ENPP1, ENPP2, NNMT, CD38 and NP. Gene ENPP1 is overly expressed in breast tumors [23], and is significantly associated with relapse-free survival upon tamoxifen treatment for recurrent disease [24]. In addition, it may be also associated with breast cancer development in an indirect way: it is a well established marker for adult obesity, which is an important risk factor for breast cancer after menopause. The protein encoded by gene ENPP2 functions as both a phosphodiesterase and a phospholipase, which catalyzes production of lysophosphatidic acid (LPA) in extracellular fluids. LPA evokes growth factor-like responses including stimulation of cell proliferation and chemotaxis. This gene product stimulates the motility of tumor cells and has angiogenic properties, and its expression is upregulated in several kinds of carcinomas. Expression of this gene is closely linked to the invasiveness of breast cancer cells [25]. It also contributes to the initiation and progression of breast cancer [26]. In addition, overexpression of ENPP2 is also associated with development and progression of prostate cancer and ovarian cancer, which suggests that it may have a fundamental role in cancer development. Gene NNMT is a novel Stat3-regulated gene and is a candidate tumor marker for various kinds of cancers, including lung cancer, colorectal cancer, bladder cancer and thyroid cancer [27]. This suggests a potential fundamental role of NNMT in cancer development. CD38 is a novel multifunctional ectoenzyme widely expressed in cells and tissues especially in leukocytes. It also functions in cell adhesion, signal transduction and calcium signaling. According to CTD [19], this gene is inferred to be associated with breast neoplasms via at least eight chemicals: alitretinoin, dacarbazine, dichlorodiphenyl, dichloroethylene, calcitriol, doxorubicin, fluorouracil, tamoxifen and tretinoin.

The Dentatorubropallidoluysian atrophy (DRPLA) pathway contains five genes: CASP1, WWp2, CASP3, INSR, and CASP7. Gene CASP1 encodes a protein which is a member of the cysteine-aspartic acid protease (caspase) family. Sequential activation of caspases plays a central role in the execution-phase of cell apoptosis. It was identified by its ability to proteolytically cleave and activate the inactive precursor of interleukin-1, a cytokine involved in processes such as inflammation, septic shock, and wound healing. It has been shown to induce cell apoptosis and may function in various developmental stages. Gene WWP2 encodes a member of the NEDD4-like protein family. It has been identified as a prognostic marker for breast cancer [28]. Gene CASP3 also encodes a protein of the caspase family. Studies have shown that CASP3 is overexpressed in a large proportion of invasive breast carcinomas [29]. Its expression is correlated with poor prognosis (higher histologic grade and high proliferation) in breast cancer patients. It may also affect response of breast tumor cell lines to chemotherapy. High levels of insulin receptor (INSR) expression in early stage breast cancers is independently and significantly associated with more favorable clinical outcomes [30]. Gene CASP7 also encodes a protein of the caspase family. Modulation of CASP7 affects response of breast tumor cell lines to chemotherapy.

### Remarks: differences and overlaps of identified pathways

Among the available pathway analysis methods, the above three have been most extensively used. Results presented in the above section suggest that the proposed approach can identify pathways significantly different from those obtained using alternatives. Although our pursuit of the biological interpretation of identified pathways is far from complete, it is already fairly clear that alternative approaches may miss important pathways.

The difference between pathways identified using the proposed method and those using the GSEA and Maxmean is dramatic. Such a finding is not surprising. For a specific pathway, both the GSEA and Maxmean analyze each gene *separately*, and then combine the gene-level analysis results to conclude the pathway-level significance. They target finding pathways that are enriched with genes marginally associated with cancer clinical outcomes. In contrast, the proposed method evaluates the joint predictive power of multiple genes in the pathways.

The proposed approach and Globaltest identify a relatively large number of common pathways. This is also not surprising. Consider the statistical framework described in the "Methods/Statistical modeling" subsection. Denote *β *as the regression coefficient in the Cox model, and *β*_0 _as the true value of *β*. The Globaltest approach tests *H*_0 _: *β*_0 _= 0 versus *H*_*A *_: *β*_0 _≠ 0. Since a necessary condition for significant predictive power is *β*_0 _≠ 0, it is reasonable that the proposed approach and Globaltest identify common pathways. On the other hand, the two approaches are not equivalent. Consider for example a hypothetical scenario with two pathways. Assume that gene expressions of the two pathways are identical. For the first pathway, assume *β*_0 _= (≠ 0). For the second pathway, assume *β*_0 _= 2. Since the Globaltest puts more emphasis on the magnitude of *β*_0_, the second pathway will be concluded as being more significant than the first one. In contrast, since the proposed method focuses on whether linear combination of genes can separate subjects into groups with different risks, the absolute magnitude is less relevant. Thus, with the proposed method, the two pathways will have an equal level of significance, as they should have.

### Evaluation of predictive power

We consider evaluating the predictive power of pathways identified using different approaches. One possibility is to follow the proposed approach and use the separation of the OPI and PPI to define predictive power. However, since the proposed approach is based on this separation, comparison of predictive power (of pathways identified using different approaches) using the OPI and PPI may not be fair.

As an alternative, we consider the following approach. (a) For each dataset and each pathway, use expressions of genes in this pathway and the K-means approach to separate subjects into two clusters; (b) Compute the logrank statistic, which is nonparametric and measures difference of survival between the two groups, and obtain the corresponding p-value; (c) For each pathway, use Fisher's approach (see the "Methods/Meta analysis" section) to combine p-values across the four studies and generate a meta analysis p-value.

With the approach described above, we investigate whether it is possible to separate subjects into two groups with different survival risks based on the patterns of gene expressions. Compared with the proposed approach, this approach is nonparametric, relies on weaker assumptions, and is more suitable for comparing different approaches. However, for a given pathway, all genes in that pathway - including noisy genes - are utilized. In addition, its nonparametric nature makes it less efficient. Thus, it is not well suited for detecting predictive pathways.

#### Gene set enrichment analysis

For the 16 pathways identified by the GSEA, the median of the meta analysis p-values obtained above is 0.897; The pathways not identified have a median (of the meta analysis p-values) of 0.108.

#### Maxmean method

The three pathways identified by the Maxmean method have a median p-value of 0.076, whereas those not identified have a median p-value of 0.143. We compare the two sets of p-values using the two-sample Wilcoxon rank sum test and obtain a p-value of 0.155, which suggests no significant difference in predictive power between pathways identified versus those not identified.

#### Globaltest method

Pathways identified by the Globaltest have a median p-value of 0.022, whereas those not identified have a median p-value of 0.223. The two-sample Wilcoxon rank sum test yields a p-value < 0.001.

#### The proposed approach

Pathways identified using the proposed approach have a median p-value of 0.014, whereas those not identified have a median p-value of 0.251. The two-sample Wilcoxon rank sum test yields a p-value < 0.001. The top 20 pathways have a median p-value of 0.007, whereas those with rank greater than 20 have a median p-value of 0.170. The two-sample Wilcoxon rank sum test yields a p-value < 0.001.

#### Remarks

Evaluation of predictive power suggests that pathways identified with the GSEA and Maxmean are not "more predictable" than those not identified. Since these two approaches focus on the marginal effects of genes, it is not surprising that they cannot detect pathways where genes have joint predictive power. In contrast, pathways identified with the Globaltest and the proposed approach have predictive power, whereas those not identified do not. The satisfactory performance of the Globaltest is also not surprising, given the considerable overlap of identified pathways with those of the proposed approach. The performance of the proposed approach is the strongest among the four approaches.

### Limitations and possible extensions

As in many other pathway analysis studies, we focus on genes with known pathway information. There is a small chance of excluding important genes. However, considering that the pathway information is accumulated from numerous independent studies, such a possibility is small. In addition, in the very near future, when pangenomic arrays become routine, this limitation may no longer be an issue. A possible alternative that uses all genes is the hybrid approach, which uses statistical clusters as a proxy for biological pathways [31]. In this study, we construct pathways using KEGG. The pathway structure may be refined if more databases are used. In some studies, researchers view the pathways as directional networks. Here, we take a simpler prospective and view the pathways as clusters of functionally related genes.

In this study, we conclude statistical significance of predictive power for a pathway if the separation between its OPI and PPI is significant. The nonparametric evaluation approach described above also assesses statistical significance. A different but related aspect that is not investigated is the *clinical significance *of predictive power (of identified gene pathways). In biomedical studies, it has been noted that, although statistical and clinical significance can be closely related, they have different implications. As in many other pathway analysis studies, we focus on detecting the statistical significance. We note that, ultimately, identified pathways needs to be evaluated in independent clinical settings to fully separate out the false positives and validate the true positives. Although our pursuit of the biological implications of identified pathways clearly shows advantage of the proposed approach, we acknowledge that our biological pursuit is still far from comprehensive.

Gene pathways are the functional units in this study. However, given our limited knowledge of pathways, we have also considered individual genes while pursing biological interpretations. We provide gene information for all pathways at the study website [32]. Pursuit of biological implications of all genes, however, is beyond scope of this study.

The goal of this study is to identify, among the many pathways, which ones have significant predictive power. Thus, we have investigated each pathway separately and compared them against each other. A related but different statistical question is to build predictive models using pathways. To solve such a problem, it would be necessary to consider the joint effects of multiple pathways. Since the study goal and statistical techniques would be significantly from those of the present study, we defer such investigations to a future study.

Heuristic theoretical justifications are provided in the "Methods" section. Since simulated gene expression data is usually significantly different from observed data [33], we have chosen not to conduct simulations here. Rather, performance of the proposed approach has been investigated using real data and also theoretically.

In the data analysis, only gene expressions are analyzed. Biomedical studies suggest that clinical and environmental risk factors may have additional predictive power. However, with the four breast cancer microarray datasets, we have failed to assemble a unified set of clinical and environmental risk factors. This poses a potential limitation to the study, and accordingly, our findings need to be explained with cautions. With other datasets, if clinical and environmental risk factors are available, the proposed method can be extended as follows. The first possible extension is to define *X *= (*X*_*clinical*_, *X*_*gene*_), where *X*_*clinical *_includes the clinical risk factors and *X*_*gene *_contains the gene expressions. We can then apply the proposed approach directly. To account for the different characteristics of clinical risk factors and gene expressions, different levels of penalties can be applied to the two sets of risk factors. *This extension can evaluate which gene pathways, together with clinical risk factors, have significant predictive power*. The second possible extension may evaluate a different aspect of gene pathways. We may first compute the OPI for the clinical risk factors and gene expressions combined. We then compute the OPI for the clinical risk factors only. We then compare the two sets of OPIs. *This extension can evaluate which pathways have significant additional predictive power beyond clinical measurements*. In this study, we focus exclusively on the linear effects of gene expressions, which is the common practice in cancer profiling studies. Following a similar strategy as in [34], the proposed approach can be extended to accommodate nonlinear gene effects. Such an extension is nontrivial and may greatly increase computational cost.

## Conclusions

Tremendous effort has been devoted to identify genomic measurements with predictive power for breast cancer prognosis. In this article, we develop a new pathway analysis method, and use it to analyze four breast cancer gene profiling studies. The proposed method advances beyond existing ones by focusing on the predictive power as opposed to estimation accuracy. It can account for the joint effects of multiple genes in pathways, and it uses multiple datasets from independent, comparable studies to improve reliability.

With the proposed method, 96 pathways are identified, many of which have a sound biological basis and have been identified as breast cancer markers in independent studies. There are also pathways that have not been previously identified. Further biomedical investigations are needed to fully understand those pathways.

## Methods

Detection of pathways with predictive power consists of the following steps.

1. (1.1) Select multiple gene profiling datasets from independent studies with *comparable designs*. The clinical aspects of the studies need to be evaluated to determine comparability. (1.2) Process each dataset separately. Normalization and imputation of missing data need to be carried out;

2. Match genes measured in different studies. Here we focus on genes measured in all studies. One possible alternative is to use all the genes and impute gene expressions not measured as zero;

3. Construct gene pathways using public databases. Only genes with known pathway information are used in downstream analysis;

4. For each dataset and each pathway, compute a statistic and corresponding p-value that can quantify the predictive power of genes within this specific pathway;

5. For each pathway, pool p-values computed from multiple datasets using Fisher's approach, and compute the overall significance level for predictive power;

6. Apply the FDR (false discovery rate) approach and identify pathways with significant predictive power.

Multiple datasets will be analyzed with the proposed approach. If studies that generate those datasets investigate the same clinical outcomes and have assembled study subjects with similar characteristics, we say *they have comparable designs*. On the other hand, they may have different experimental settings. Particularly, they may use different platforms for profiling. Studies with comparable designs can be pooled for meta analysis. However, when the experimental settings are not comparable, estimates generated from one study cannot be used to make predictions for subjects in the other studies.

Steps 1-3 will be carried out using well-developed existing approaches. We refer to the published literature [15,16] and the "Results and Discussion" section for data selection, data processing, gene matching, and pathway construction. In the following subsections, we provide detailed descriptions of Steps 4-6.

### Quantification of the predictive power of a single pathway

In this subsection, we consider a single dataset and a single pathway, and describe how to quantify its predictive power.

#### Statistical modeling

Consider a pathway composed of *m *genes. Denote *X *= (*X*^1^, ..., *X*^*m*^) as the gene expressions. Denote *U *and *V *as the relapse and censoring time, respectively. Under right censoring, one observation consists of (*T *= *min*(*U*, *V*), Δ = *I*(*U *≤ *V*), *X*). We assume the Cox proportional hazards model, where(1)

Here *λ*_0_(*u*) is the unknown baseline hazard and *β *is the length *m *regression coefficient. Assume *n *i.i.d. observations: (*T*_*i*_, *δ *_*i*_, *X*_*i*_); *i *= 1 ... *n*. The log-partial likelihood function is:(2)

where *r*_*j *_= {*k*: *T*_*k *_≥ *T*_*j*_} is the risk set at time *T*_*j*_.

#### Penalized estimation

Penalization has been extensively used as a regularized estimation tool in cancer genomic studies [35]. With cancer genomic data, it is common that the sizes of some gene pathways are comparable to or even larger than the sample size. For the four datasets we analyze, the smallest sample size is 58, and the largest pathway has 51 genes. With large pathways, direct maximization of the log-likelihood function may lead to unreliable or multiple maximizers. Penalization can regularize the maximizer, making it "regular" and unique. In addition, pathways defined in databases such as KEGG, BioCarta, and GO are not tailored to any specific disease clinical outcomes. Thus, even in a predictive pathway, there may still exist noisy genes. Penalization can select predictive genes. Using only predictive genes can be more informative than using all of the genes.

We propose estimating *β *with(3)

where *λ*_*n *_is the data-dependent tuning parameter, *β*_*j *_is the *j*th component of *β*, and 0 <*γ *< 1 is the fixed penalization parameter.  defined in (3) is a *bridge penalized estimate *[36,37]. In a recent study, Huang et al. [37] established that the bridge penalized estimate has the "oracle" estimation and selection properties and performs better than many alternative penalization methods.

#### Determination of the significance of predictive power

Consider a single dataset with *n *subjects. For a pathway composed of *m *genes, the significance of its predictive power can be computed as follows.

1. Compute the *Observed Predictive Index *(OPI).

(a) Randomly partition the data into a training set and a testing set with sizes 2*n*/3 and *n*/3, respectively;

(b) Compute  defined in (3) using only subjects in the training set;

(c) For subjects in the testing set, compute the predictive risk scores '*X *using the training set estimate. Dichotomize those scores at the median and create two risk groups. Compute the logrank statistic that measures the difference of survival between the two groups;

(d) Repeat Steps (a)-(c) *B *(e.g. 100) times. The *B *logrank statistics will be referred to as the OPI.

2. Compute the *Permuted Predictive Index *(PPI), which serves as the reference distribution for the OPI. The PPI is computed in a similar manner as the OPI. The only difference is that, prior to each partition, the survival time and event indicator (*T*, Δ) are randomly permuted (and then coupled with gene expressions).

3. Conduct a two-sample Wilcoxon rank sum test of the OPI versus the PPI. The resulting p-value measures the significance of predictive power.

In Step 1(a), we "create" independent datasets using partitions. As discussed above, even for studies with comparable designs, their experimental settings may not be comparable. To make the proposed method broadly applicable, we use random partitions to guarantee the comparability of training and testing sets. The random split also closely mimics the 0.632 bootstrap [38]. In Step 1(b), we estimate the best linear combination of genes. In Step 1(c), we quantify the predictive power of genes, or more accurately their linear combination '*X*. In cancer survival analysis, the logrank statistic has been extensively used as a measure of predictive power [39]. For simplicity and interpretability, only two risk groups are created and the two-sample logrank statistic is computed. Possible alternatives to the two-sample logrank statistic include the multi-sample logrank statistic, the logrank statistic for a continuous marker, and the supremum versions of the aforementioned statistics [40,41]. Under certain situations, the alternative statistics can be more powerful at the cost of increased computational complexity. Due to the risk of an extreme partition, a single partition and a single logrank statistic may not be sufficient. Thus, in Step 1(d), we generate multiple logrank statistics via multiple partitions.

The standardized and squared logrank statistics generated in Step 1 are asymptotically *χ*^2 ^distributed. In cancer genomic studies, the sample sizes are often small. It is not clear how precise the asymptotic *χ*^2 ^approximation is in these settings. Thus, in Step 2, we use permutations to generate the reference distribution of the OPI.

The two-sample Wilcoxon rank sum test in Step 3 measures how well the OPI and PPI are separated. Clear separation of the OPI and PPI indicates that *the linear combinations of genes in this pathway are capable of separating patients into groups with different survival risks*. Thus, a significant p-value from the Wilcoxon test suggests significant predictive power of this pathway.

### Meta analysis

In a single dataset and for a specific pathway, the procedure described above can generate a p-value that measures its predictive power. For many cancer clinical outcomes, there exist multiple studies with comparable designs [2]. As shown in [15,16] and others, meta analysis of multiple datasets can generate more reliable results than analysis of a single dataset.

Assume there are *D *datasets from independent studies with comparable designs. For a specific pathway, we first analyze each dataset separately using the approach described above. Denote *p*_1 _... *p*_*D *_as the *D *p-values generated from the *D *datasets. With Fisher's approach, the pooled statistic is , which is *χ*^2 ^distributed with degrees of freedom 2*D *[42]. The p-value of *s*, denoted as , measures the significance of predictive power concluded from the *D *datasets.

One potential drawback of Fisher's approach is that the combined level of significance may be seriously affected by a small number of extreme values. In our data analysis, we examine the p-values across the four studies and find that the significance levels are pretty "uniform" (results provided in the Additional File 1).

If, with other datasets, significantly varying p-values are observed, alternative meta analysis approaches may be needed.

### Controlling the FDR

Assume there are a total of *N *pathways. Denote  as the *N *p-values generated using Fisher's approach. We use the following approach to control the FDR. (a) Set the target FDR to *q *= 0.2; (b) Order the p-values ; (c) Let *r *be the largest *i *such that  ≤ *i*/*N *× *q*/*c*(*N*); (d) Pathways corresponding to  are concluded as having significant predictive power.

Different pathways may share common genes, since one gene may have multiple biological functions. To account for possibly complicated correlations among p-values caused by overlapping pathways, we set [43].

### Asymptotic considerations

Consider a single dataset and a single pathway. Under the Cox model, detection of the predictive power amounts to properly estimating *β *and determining its predictive power. Denote *β*_0 _as the true value of *β*. When *β*_0 _= 0, genes in this pathway are not associated with survival, and this pathway has no predictive power. When *β*_0 _≠ 0, this pathway is predictive, where the predictive power can be measured with the logrank statistic [44]. Following [37], under one of the following two conditions,  defined in (3) is a consistent estimate of *β*_0_:

1. *n *→ ∞ and *m *= *o*(*n*^1/2^);

2. *n *→ ∞ and *m *= *o*(exp(*n*^*α*^)), where *α *is a fixed constant that depends on *X*. In addition, *l *= *o*(*n*^1/2^), where *l *is the number of nonzero components of *β*_0_. Moreover, the irrepresentable condition in [45] must be satisfied.

With  being a consistent estimate of *β*_0_, validity of the p-value from the Wilcoxon rank sum test follows from validity of the logrank statistic and permutation test. In addition, validity of the meta analysis using Fisher's approach has been discussed in [42] and references therein.

Consider *N *pathways and their p-values . To control the FDR, uniform consistency of the p-values is needed. For a specific pathway, the consistency has been discussed above. However, consistency for each individual pathway does not automatically lead to uniform consistency. As shown in [46], uniform consistency further requires that log(*N*)/*n *→ 0, as *n *→ ∞.

## Remarks

We have described the proposed approach in the context of cancer prognosis studies using microarrays. With minor modification, the approach is also applicable to other disease clinical outcomes and other profiling platforms. For example, for diagnosis studies with binary outcomes, the Cox model can be replaced with the logistic model, and the logrank statistic can be replaced with the classification error. The remaining components of the proposed approach will then be applicable.

## Authors' contributions

Both authors were involved in the study design, data analysis, and writing. Both authors read and approved the final manuscript.

## Supplementary Material

Additional file 1 **Predictive pathways identified using the proposed and alternative approaches**. This file contains information on all the pathways identified using the proposed and alternative approaches. Click here for file
